# Association Between Sedentary Behavior, Physical Activity, and Obesity: Inactivity Among Active Kids

**Published:** 2008-12-15

**Authors:** Scott T. Leatherdale, Suzy Wong

**Affiliations:** Department of Population Studies and Surveillance, Cancer Care Ontario. Dr Leatherdale is also affiliated with the Department of Health Studies and Gerontology, University of Waterloo, Waterloo, Ontario, Canada; and the Dalla Lana School of Public Health, University of Toronto, Toronto, Ontario, Canada; University of Waterloo, Waterloo, Ontario, Canada

## Abstract

**Introduction:**

Sedentary behavior and physical activity are not mutually exclusive behaviors. The relative risk of overweight for adolescents who are highly sedentary and highly physically active is unclear. A better understanding of the relationship between sedentary behaviors, physical activity, and body mass index (BMI) would provide insight for developing interventions to prevent or reduce overweight.

**Methods:**

Using the physical activity module of the School Health Action, Planning and Evaluation System (SHAPES), we collected data from 25,060 students in grades 9 through 12 from 76 secondary schools in Ontario, Canada. Sex-specific logistic regression analyses were performed to examine how BMI, weight perceptions, social influences, team sports participation, and smoking behavior were associated with being 1) high active-high sedentary, 2) low active-low sedentary, and 3) low active-high sedentary.

**Results:**

Low active-high sedentary boys were more likely to be overweight than high active-low sedentary boys (adjusted odds ratio [AOR], 1.60; 95% confidence interval [CI], 1.01-2.58). When compared with high active-low sedentary girls, girls who were low active-high sedentary (OR, 2.24; 95% CI, 1.23-4.09) or high active-high sedentary (OR, 1.91; 95% CI, 1.01-3.61) were more likely to be overweight.

**Conclusion:**

Sedentary behavior may moderate the relationship between physical activity and overweight. Developing a better understanding of sedentary behavior in relation to physical activity and overweight is critical for preventing and reducing overweight among youth.

## Introduction

The prevalence of overweight among children and adolescents has increased markedly in the last 2 decades in Canada and the United States (1). In 2004, 26% of Canadian youth (2) and 34% of American youth were overweight (3). The high prevalence of overweight among youth is cause for concern because overweight youth are almost twice as likely as normal-weight youth to become overweight adults (4). Considering that overweight is associated with an increased risk of cardiovascular disease, stroke, type 2 diabetes, and some types of cancer (5-7), reducing the prevalence of overweight among youth is justifiably a public health priority.

The rapid increase in the prevalence of childhood obesity during the past 2 decades suggests that environmental factors may play a greater role than genetic factors (8). The increased prevalence of obesity is suspected to be the result of reduced energy expenditure or increased energy intake (9). Many organizations have developed recommendations regarding the amount of time children and adolescents should participate in physical activities. Canada's *Physical Activity Guides for Children and Youth* recommend that children and adolescents should accumulate at least 90 minutes of moderate-intensity to vigorous-intensity physical activity per day (10), whereas American guidelines recommend that children and adolescents participate in at least 60 minutes of moderate-intensity physical activity most days of the week, preferably daily (11).

Sedentary behavior is emerging as an important component of obesity and should be recognized as behavior that is distinct from physical activity (12,13). Defining sedentary behavior as the absence of physical activity fails to acknowledge the range and complexity of sedentary behavior (ie, watching television, playing video games, using the computer, reading, and doing homework each may have different implications for obesity). National organizations have developed recommendations regarding the amount of time that children and adolescents are sedentary. Canada's *Physical Activity Guides for Children and Youth* recommend that children and adolescents decrease by at least 90 minutes per day the amount of time spent in nonactive activities, such as watching television, watching videos, and sitting at a computer (10), and the American Academy of Pediatrics recommends that children's total media time (with entertainment media) be limited to no more than 1 to 2 hours of quality programming per day (14).

Theoretically, youth could be considered both highly active and highly sedentary. For instance, over the course of the day, youth have sufficient time to both perform more than 90 minutes of moderate-intensity to vigorous-intensity physical activity (*highly active*) and spend more than 2 hours in sedentary activities (*highly sedentary*), particularly on weekends. Similarly, youth may fit into other unique subgroups, such as those who are highly active and low sedentary, low active and low sedentary, or low active and highly sedentary. Although previous research has found that high levels of physical activity are not mutually exclusive with high levels of sedentary behavior (15), the relative risk of overweight for each different group should be examined. This new understanding may provide insight for tailoring interventions to prevent or reduce overweight among youth.

We sought to determine 1) the extent to which the 4 groups (high active-low sedentary, high active-high sedentary, low active-low sedentary, low active-high sedentary) exist, 2) the relative risk of overweight for each group, and 3) characteristics that predict being in a group.

## Methods

### Design

We used a cross-sectional survey design and self-reported data from students in grades 9 through 12 from 76 secondary schools in Ontario, Canada, that were collected in 2005-2006 as part of the School Health Action, Planning and Evaluation System (SHAPES). The physical activity and tobacco modules of SHAPES were administered to consenting students (in each school, 50% of classes completed the physical activity module, and 50% completed the tobacco module); however, only data from the physical activity module were used in this study. The physical activity module asked students about physical and sedentary activity patterns, height and weight, correlates for physical and sedentary activities, enabling factors specific to physical activity within schools, social influences, beliefs about opportunities for physical activity offered within the school environment, and smoking behavior. Testing using Spearman correlations for self-reported measures for height (*r* = 0.97, *P* < .001), weight (*r* = 0.98, *P* < .001), and physical activity (*r* = 0.44, *P* < .01) previously determined significant criterion validity (16). Additional details about SHAPES and the different modules and their psychometric properties are available in print (16-18) and online (www.shapes.uwaterloo.ca).

### Data collection

All surveys were completed during class time, and participants were not provided compensation. Active information with passive consent was used to reduce demands on schools and to increase student participation rates. Parents were mailed a letter that described the study. If parents wanted their children to be removed from the study, they were instructed to call a toll-free telephone number or submit a signed form. The University of Waterloo Office of Research Ethics and the school boards and public health ethics committees of participating schools approved all procedures, including passive consent.

### Participants

Of the 34,578 eligible students selected to complete the physical activity module, 73.5% (n = 25,416) completed the survey; missing respondents resulted from absenteeism on the day of the survey and from parent or student refusal. This distribution is consistent with previous SHAPES data collections (19,20).

### Measures

Sedentary behavior was measured by asking respondents to report the number of hours of screen time spent for each day of the week (ie, time spent watching television or movies, playing video or computer games, surfing the Internet, or instant messaging). Average screen time per day was calculated on the basis of the average time reported during the previous week, and responses were coded into 3 categories (<1 hour per day, 1-3 hours per day, >3 hours per day). Students with less than 1 hour of screen time per day were classified as low sedentary, and students with more than 3 hours per day were classified as high sedentary.

Units used to measure respondents' physical activity rates were kilocalories per kilogram of body weight per day (KKD). Physical activity was measured by asking respondents how many minutes of vigorous-intensity physical activity (VPA) (ie, physical activities that increase your heart rate and make you breathe hard and sweat, such as jogging or team sports) and moderate-intensity physical activity (MPA) (ie, lower-intensity physical activities, such as walking or biking to school) they engaged in on each of the last 7 days. The average KKD expended in VPA and MPA were calculated as follows: KKD = [(hours of VPA x 6 MET) + (hours of MPA x 3 MET)] / 7 days. This calculation assumes that the standard metabolic equivalent (MET), a unit used to estimate the amount of oxygen used by the body during physical activity, for VPA was 6 and for MPA was 3 (21).

Although adolescents tend to substantially overreport time spent doing physical activity (16,22), the SHAPES measures are valid for differentiating students who report less time doing physical activity from students who report more time doing physical activity (17). Therefore, rather than using predetermined cutpoints (eg, <3 KKD, 6-8 KKD) to classify students’ physical activity levels, it is more meaningful to compare the relative physical activity levels of students within the sample (16). Therefore, within our sample, students who were 1 SD below (≤16th percentile) the sample mean for KKD were classified as low active, students 1 SD above (≥84th percentile) the sample mean for KKD were classified as high active, and students within 1 SD (17th-83rd percentile) of the sample mean for KKD were classified as moderately active. Students were then grouped into 4 behavioral categories on the basis of their sedentary behavior and physical activity scores: 1) high active-low sedentary (control group), 2) high active-high sedentary, 3) low active-low sedentary, and 4) low active-high sedentary.

Body mass index (BMI, kg/m^2^) was calculated by using previously validated (16) self-reported measures of weight and height. Students who were less than the 5th percentile for BMI by sex were classified as underweight, students who were in the 85th percentile or higher for BMI by sex were classified as at risk of overweight/overweight, and students who were in the 5th to less than the 85th percentile for BMI by sex were classified as normal weight (21). Students were asked to report their perception of their body weight (overweight, about the right weight, underweight); the physical activity level of their father and mother (active or somewhat active vs inactive); whether their parent(s) encourage physical activity (encourage vs do not encourage) or are supportive of their child's participation in physical activity (supportive vs unsupportive); how many of their 5 closest friends are physically active (0, 1-2, ≥3); and whether they participate in intramural teams at school (yes/no), varsity teams at school (yes/no), or team sports outside of school (yes/no). The smoking stage categories used were consistent with existing research that daily smokers had smoked every day or almost every day in the 30 days preceding the survey, occasional smokers had smoked some days or only 1 or 2 days in the 30 days preceding the survey, and nonsmokers had smoked fewer than 100 cigarettes in their lifetimes and had not smoked at all in the last 30 days. The location of the school a student attended was also recorded (rural, suburban, inner city/urban).

### Analyses

We calculated the prevalence of each of the 4 behavioral categories within the study sample by sex. Using the sample of students who were in these groups, we then calculated the sex-specific prevalence of sample characteristics (grade, social influences for physical activity, smoking, BMI and perceptions of weight, sports team participation, and school location)We calculated the prevalence of each of the 4 behavioral categories within the study sample by sex. Using the sample of students who were in these groups, we then calculated the sex-specific prevalence of sample characteristics (grade, social influences for physical activity, smoking, BMI and perceptions of weight, sports team participation, and school location) by the 4 behavioral categories. Sex-specific logistic regression analyses were used to examine how the 4 different behavioral categories were associated with being overweight relative to being normal weight or underweight. Sex-specific logistic regression analyses were performed to examine how BMI, weight perceptions, social influences, team sports participation, and smoking behavior were associated with being 1) high active-high sedentary, 2) low active-low sedentary, and 3) low active-high sedentary, relative to being high active-low sedentary. Each logistic regression analysis performed used the proc genmod command with school as a class statement to control for the effect of clustering of the behaviors within schools. We also controlled for age, grade, and school location in all analyses. We used SAS version 8.02 (SAS Institute, Inc, Cary, North Carolina) for all analyses.

## Results

Data were available for 25,060 students in the 76 schools. The sample was 51.1% (n = 12,806) male and 48.9% (n = 12,254) female. Students' average age was 15.5 (standard deviation [SD], 1.2) years. Overall, boys were older than girls (χ^2^ = 68.82, *df* = 5, *P* < .001). For smoking behaviors, 2,302 (9.5%) students were classified as daily smokers, 2,178 (9.0%) were classified as occasional smokers, and 19,665 (81.5%) were classified as nonsmokers. Among boys, the mean BMI was 22.0 (SD, 3.54) kg/m^2^, and 13.0% were classified as overweight. Among girls, the mean BMI was 21.3 (SD, 3.40) kg/m^2^, and 13.3% were classified as overweight. Average screen time per day was 2.7 (SD, 1.7) hours for all students.

A total of 4,066 (16.3%) students were classified as low active, 17,183 (68.7%) were classified as moderately active, and 3,751 (15.0%) were classified as highly active; 2,538 (10.3%) students were classified as low sedentary, 14,180 (57.3%) as moderately sedentary, and 8,020 (32.4%) as high sedentary. A total of 3,609 students could be classified into 1 of the 4 behavioral categories being examined, and significant sex differences among groups existed (χ^2^ = 173.87, *df* = 3, *P* < .001). Overall, 226 (10.9%) boys and 162 (10.6%) girls were high active-low sedentary, 866 (41.6%) boys and 385 (25.2%) girls were high active-high sedentary, 132 (6.3%) boys and 268 (17.6%) girls were low active-low sedentary, and 859 (41.2%) boys and 711 (46.6%) girls were low active-high sedentary.

More boys in grade 12 were low active-high sedentary than students in lower grades (*P* < .001), yet the prevalence of being high active-low sedentary remained stable after grade 9 (Table 1). Most boys involved in intramural teams at school (*P* < .001), varsity teams at school (*P* < .001), or sports teams in the community (*P* < .001) were high active-high sedentary.

Most overweight girls were low active-high sedentary (*P* < .001), as were most girls who perceived themselves as being overweight (*P* < .001) (Table 2). Similar to boys, most girls involved in intramurals at school (*P* < .001), varsity teams at school (*P* < .001), or sports teams in the community (*P* < .001) were high active-high sedentary.

### Risk of overweight

When compared with high active-low sedentary boys, boys who were low active-high sedentary were more than 1.5 times more likely to be overweight (Table 3). When compared with high active-low sedentary girls, girls who were low active-high sedentary (adjusted odds ratio [AOR], 2.24) or high active-high sedentary (AOR, 1.91) were more likely to be overweight.

### Factors associated with behavioral categories among boys

Among boys, a perception of being overweight was the only factor associated with being high active-high sedentary (AOR, 2.17) (Table 4). Being underweight (AOR, 2.63), a perception of being overweight (AOR, 3.05), and having parents who encourage participation in physical activity (AOR, 2.52) were positively associated with being low active-low sedentary. However, having parents who are supportive of physical activity (AOR, 0.20) and participating in intramurals at school (AOR, 0.31), varsity teams at school (AOR, 0.45), or teams in the community (AOR, 0.32) were negatively associated with being low active-low sedentary. Similarly, being underweight (AOR, 2.44) or having a perception of being overweight (AOR, 3.72) was positively associated with being low active-high sedentary. However, participating in intramurals at school (AOR, 0.38), varsity teams at school (AOR, 0.40), or sports teams in the community (AOR 0.20) was negatively associated with being low active-high sedentary.

### Factors associated with behavioral categories among girls

Among girls, a perception of being overweight was positively associated with being high active-high sedentary (AOR, 1.82), and participating in a team sport outside of school was negatively associated with being high active-high sedentary (AOR, 0.34) (Table 5). Being underweight was positively associated with being low active-low sedentary (AOR, 2.64), whereas participating in intramurals at school (AOR, 0.38) or sports teams in the community (AOR, 0.10) was negatively associated with being low active-low sedentary. A perception of being overweight was positively associated with being low active-high sedentary (AOR, 1.99), and participating in intramurals at school (AOR, 0.48) or sports teams in the community (AOR, 0.09) was negatively associated with being low active-high sedentary.

## Discussion

This study characterized 4 subpopulations of youth in relation to physical activity and sedentary behavior patterns. The most prevalent group consisted of boys and girls who were considered both highly active and highly sedentary. This new insight is consistent with the results of previous research, which observed that youth tended to cluster into groups, of which members of one performed high levels of physical activity and spent a considerable amount of time playing video games and watching television (15,23). These findings support the notion that being highly sedentary is not equivalent to a lack of physical activity and that subgroups of highly active-highly sedentary youth do exist. Future studies should not assume that youth who spend substantial amounts of time performing sedentary behaviors are not also spending substantial amounts of time performing physical activity.

We also found sex differences across groups. A higher proportion of boys than girls were high active-high sedentary, whereas a higher proportion of girls than boys were low active-low sedentary. These findings are inconsistent with results of one study, which found that more girls and fewer boys than expected were found in the cluster characterized by high levels of watching television or videos and sitting while listening to music or talking on the telephone and doing homework (23).

As expected, low active-high sedentary boys and girls were more likely to be overweight than were high active-low sedentary boys and girls. However, high active-high sedentary girls were more likely to be overweight than were high active-low sedentary girls. This finding suggests that the levels of physical activity that the high active girls engaged in may be insufficient to attenuate the negative health consequences of the high levels of sedentary activities in which they engaged. These findings suggest that the relationship between BMI and physical activity may be moderated by sedentary activity, a consideration for practitioners. Thus, considering levels and types of sedentary activity when trying to understand the relationship between BMI and physical activity is essential. This may explain why some researchers (24,25) did not find an association between BMI and physical activity in children and adolescents, despite the postulated relationship between reduced energy expenditure and obesity (9). One meta-analysis (26), which found that sedentary behavior was not associated with physical activity or BMI, also suggests that future research needs to consider different types and levels of sedentary behaviors when examining associations between physical activity and obesity.

An understanding of the characteristics that predict being in a behavioral group may contribute to the future development and targeting of obesity prevention initiatives beyond understanding the correlates of physical activity and sedentary behavior. For instance, among both boys and girls, participating in intramural teams was negatively associated with being in the low active groups, whereas a perception of being overweight was associated with being in the high sedentary groups. Program planners should provide additional prevention resources to schools that have lowest rates of intramural participation among students or to students who perceive that they are overweight, rather than just the students who are considered overweight. Intensive prevention programs could be implemented in schools that are putting students at the greatest risk for physical inactivity, if these "high-risk" schools are specifically targeted. A similar approach could be used for targeting students who may be at high risk. Such targeting could help extend limited education and public health funds for intervention by reducing the number of schools that require intensive intervention or by tailoring programs to student populations where they are most likely to work. However, considering that sex differences in student characteristics predicted being in a behavioral group, tailoring resources to the unique needs of subgroups of boys and girls may be needed.

This study has several limitations. The cross-sectional nature of the data prevents causal inferences to be made. For example, a perception of being overweight may lead to being in high sedentary groups, or being in high sedentary groups may lead to a perception of overweight. Because no data on ethnicity or socioeconomic status were available, we were unable to examine whether sedentary behavior or physical activity vary by ethnic groups or socioeconomic strata. Another limitation was our use of self-reported data. However, the questionnaire has previously demonstrated satisfactory reliability and validity (16), and honest reporting was encouraged by ensuring confidentiality during data collection. The sedentary behaviors considered in this manuscript were limited to screen-based behaviors. Future studies should broaden the scope of sedentary behaviors considered (eg, time spent commuting). Because a large amount of data were excluded as a result of characterizing the sample into different groups and because the groups were not determined using cluster analysis, our findings should not be generalized to all students. Despite these limitations, our findings contribute to our understanding of the relationship between physical activity, sedentary behavior, and overweight among youth.

Our results demonstrate that high levels of physical activity and high levels of sedentary behavior among adolescents are not mutually exclusive. Furthermore, the relative risk of overweight differed significantly between behavioral groups. This finding suggests that levels of both physical activity and sedentary behavior should be considered when trying to understand the factors associated with overweight and in the development of effective obesity prevention initiatives. Interventions to reduce obesity by increasing physical activity levels may not be effective if levels of sedentary behavior remain high. Further research using longitudinally measured physical activity and sedentary behavior patterns would provide valuable insight into determining the amounts of physical activity required to prevent obesity at varying levels of sedentary behavior. Improving our understanding of the factors associated with being in different physical activity and sedentary behavior groups may contribute to identifying subgroups to target for obesity interventions. Further research is required to develop effective strategies for motivating youth to become highly active and to discourage them from being sedentary.

## Figures and Tables

**Table 1:** Characteristics of Boys (N = 2,083) by Behavioral Category, School Health Action, Planning and Evaluation System (SHAPES), Ontario, Canada, 2005-2006

Characteristic	High Active-Low Sedentary (n = 226), No.^a^ (%)	High Active-High Sedentary (n = 866), No.^a^ (%)	Low Active-Low Sedentary (n = 132), No.^a^ (%)	Low Active-High Sedentary (n = 859), No.^a^ (%)	χ^2^, *df*	*P* Value
**Grade level**						
9	88 (15.1)	278 (47.5)	27 (4.6)	192 (32.8)	66.7, 9	<.001
10	52 (8.9)	266 (45.6)	34 (5.9)	231 (39.6)		
11	45 (9.8)	183 (39.6)	27 (5.8)	207 (44.8)		
12	41 (9.0)	139 (30.7)	44 (9.7)	229 (50.6)		
**Father's physical activity level**						
Inactive	19 (6.4)	84 (28.6)	27 (9.2)	164 (55.8)	44.1, 3	<.001
Active/somewhat active	201 (12.2)	717 (43.7)	97 (5.9)	627 (38.2)		
**Mother's physical activity level**						
Inactive	33 (7.7)	137 (32.0)	27 (6.3)	231 (54.0)	40.4, 3	<.001
Active/somewhat active	187 (11.9)	696 (44.4)	98 (6.3)	586 (37.4)		
**Parents' encouragement of physical activity**						
Do not encourage	38 (6.7)	151 (26.4)	39 (6.8)	344 (60.1)	125.8, 3	<.001
Encourage	188 (12.5)	710 (47.3)	93 (6.2)	510 (34.0)		
**Parents' support of physical activity**						
Unsupportive	8 (3.1)	56 (21.4)	19 (7.3)	178 (68.2)	101.7, 3	<.001
Supportive	218 (12.1)	807 (44.8)	110 (6.1)	665 (37.0)		
**No. of close friends who are active**						
0	5 (2.1)	28 (14.4)	25 (12.9)	137 (70.6)	304.2, 6	<.001
1-2	15 (4.1)	76 (20.5)	36 (9.7)	244 (65.8)		
≥3	207 (13.8)	761 (50.5)	68 (4.5)	469 (31.2)		
**Smoking status^b^**						
Nonsmoker	20 (8.0)	108 (43.6)	14 (5.7)	106 (42.7)	5.6, 6	.47
Occasional smoker	26 (14.6)	70 (39.3)	13 (7.3)	69 (38.8)		
Daily smoker	165 (10.8)	642 (42.0)	88 (5.8)	633 (41.4)		
**Body mass index^c^**						
Underweight	32 (7.4)	142 (32.9)	41 (9.5)	217 (50.2)	53.5, 6	<.001
Normal weight	172 (12.4)	628 (45.4)	75 (5.4)	508 (36.8)		
Overweight	22 (8.2)	96 (35.8)	16 (6.0)	134 (50.0)		
**Perception of body weight**						
Overweight	21 (4.4)	156 (32.8)	36 (7.6)	262 (55.2)	89.1, 6	<.001
Right weight	163 (14.4)	523 (46.3)	58 (5.2)	385 (34.1)		
Underweight	42 (9.1)	182 3(39.6)	33 (7.2)	203 (44.1)		
**Participate in intramural teams at school**						
No	96 (7.1)	417 (30.7)	100 (7.3)	746 (54.9)	376.0, 3	<.001
Yes	129 (18.9)	437 (63.9)	24 (3.5)	94 (13.7)		
**Participate in varsity teams at school**						
No	84 (6.5)	394 (30.6)	98 (7.6)	713 (55.3)	360.8, 3	<.001
Yes	140 (18.6)	462 (61.5)	26 (3.5)	123 (16.4)		
**Participate in team sports outside of school**						
No	57 (5.1)	295 (26.4)	90 (8.0)	676 (60.5)	460.0, 3	<.001
Yes	168 (18.1)	561 (60.4)	35 (3.8)	164 (17.7)		

a Numbers may not add to total because of missing values. All analyses controlled for age, grade level, and school location.

b Daily smokers had smoked every day or almost every day in the 30 days preceding the survey, occasional smokers had smoked some days or only 1 or 2 days in the 30 days preceding the survey, and nonsmokers had smoked fewer than 100 cigarettes in their lifetimes and had not smoked at all in the last 30 days.

c Students who were less than the 5th percentile for BMI by sex were classified as underweight, students who were in the 85th percentile or higher for BMI by sex were classified as at risk of overweight/overweight, and students who were in the 5th to less than the 85th percentile for BMI by sex were classified as normal weight.

**Table 2:** Characteristics of Girls (N = 1,526) by Behavioral Category, School Health Action, Planning and Evaluation System (SHAPES), Ontario, Canada, 2005-2006

Characteristic	High Active-Low Sedentary (n = 162), No.^a^ (%)	High Active-High Sedentary (n = 385), No.^a^ (%)	Low Active-Low Sedentary (n = 268), No.^a^ (%)	Low Active-High Sedentary (n = 711), No.^a^ (%)	χ^2^, *df*	*P* Value
**Grade level**						
9	63 (15.0)	161 (38.3)	46 (11.0)	150 (35.7)	130.0, 9	<.001
10	28 (6.8)	113 (27.2)	60 (14.4)	214 (51.6)		
11	41 (12.0)	57 (16.6)	57 (16.6)	188 (54.8)		
12	30 (8.6)	54 (15.5)	105 (30.2)	159 (45.7)		
**Father's physical activity level**						
Inactive	11 (4.4)	37 (14.9)	60 (24.1)	141 (56.6)	39.1, 3	<.001
Active/somewhat active	143 (12.4)	317 (27.5)	192 (16.7)	499 (43.4)		
**Mother's physical activity level**						
Inactive	22 (6.8)	50 (15.3)	62 (19.0)	192 (58.9)	37.5, 3	<.001
Active/somewhat active	138 (11.7)	331 (28.2)	204 (17.4)	501 (42.7)		
**Parents' encouragement of physical activity**						
Do not encourage	21 (4.6)	72 (15.6)	97 (21.0)	271 (58.8)	72.3, 3	<.001
Encourage	141 (13.3)	311 (29.3)	171 (16.1)	439 (41.3)		
**Parents' support of physical activity**						
Unsupportive	6 (2.9)	34 (16.3)	42 (20.1)	127 (60.7)	32.9, 3	<.001
Supportive	156 (11.9)	348 (26.6)	224 (17.2)	579 (44.3)		
**No. of close friends who are active**						
0	5 (5.6)	14 (11.2)	21 (16.8)	83 (66.4)	159.9, 6	<.001
1-2	21 (4.9)	53 (12.3)	93 (21.6)	263 (61.2)		
≥3	139 (14.6)	318 (33.3)	147 (15.4)	351 (36.7)		
**Smoking status^b^**						
Nonsmoker	14 (7.1)	56 (28.3)	32 (16.1)	96 (48.5)	6.1, 6	.41
Occasional smoker	11 (8.1)	40 (29.4)	24 (17.6)	61 (44.9)		
Daily smoker	127 (11.2)	274 (24.2)	200 (17.7)	531 (46.9)		
**Body mass index^c^**						
Underweight	18 (6.1)	71 (24.0)	55 (18.6)	152 (51.3)	24.3, 6	<.001
Normal weight	131 (13.1)	260 (25.9)	177 (17.6)	435 (43.4)		
Overweight	13 (5.8)	52 (23.5)	36 (16.0)	123 (54.7)		
**Perception of body weight**						
Overweight	26 (5.4)	119 (24.9)	78 (16.3)	255 (53.4)	30.7, 6	<.001
Right weight	144 (13.7)	212 (25.5)	154 (18.5)	352 (42.3)		
Underweight	19 (9.9)	52 (27.1)	26 (13.5)	95 (49.5)		
**Participate in intramural teams at school**						
No	85 (7.2)	226 (19.2)	231 (19.7)	633 (53.9)	217.3, 3	<.001
Yes	77 (23.0)	157 (47.0)	30 (9.0)	70 (21.0)		
**Participate in varsity teams at school**						
No	70 (6.4)	205 (18.7)	221 (20.2)	599 (54.7)	220.5, 3	<.001
Yes	92 (22.4)	178 (43.4)	40 (9.8)	100 (24.4)		
**Participate in team sports outside of school**						
No	35 (3.4)	169 (16.5)	218 (21.4)	600 (58.7)	382.2, 3	<.001
Yes	127 (26.0)	214 (43.8)	45 (9.2)	103 (21.0)		

a Numbers may not add to total because of missing values. All analyses controlled for age, grade level, and school location.

b Daily smokers had smoked every day or almost every day in the 30 days preceding the survey, occasional smokers had smoked some days or only 1 or 2 days in the 30 days preceding the survey, and nonsmokers had smoked fewer than 100 cigarettes in their lifetimes and had not smoked at all in the last 30 days.

c Students who were less than the 5th percentile for BMI by sex were classified as underweight, students who were in the 85th percentile or higher for BMI by sex were classified as at risk of overweight/overweight, and students who were in the 5th to less than the 85th percentile for BMI by sex were classified as normal weight.

**Table 3:** Adjusted Odds of Being Overweight, by Behavioral Category, Male (n = 2,083) and Female (n = 1,526) Students, School Health Action, Planning and Evaluation System (SHAPES), Ontario, Canada, 2005-2006

Behavioral Category	Overweight Boys(n = 268) vs Normal Weight and Underweight^a^ Students (n = 1,815), AOR^b^ (95% CI)	Overweight Girls (n = 225) vs Normal Weight and Underweight^a^ Students (n = 1,299)^c^, AOR^b^ (95% CI)
High active-low sedentary	1 [Reference]	1 [Reference]
High active-high sedentary	1.15 (0.71-1.88)	1.91 (1.01-3.61)^d^
Low active-low sedentary	1.16 (0.58-2.30)	1.53 (0.78-2.99)
Low active-high sedentary	1.60 (1.01-2.58)^e^	2.24 (1.23-4.09)^f^

Abbreviations: AOR, adjusted odds ratio; CI, confidence interval.

a Students who were less than the 5th percentile for BMI by sex were classified as underweight, students who were in the 85th percentile or higher for BMI by sex were classified as at risk of overweight/overweight, and students who were in the 5th to less than the 85th percentile for BMI by sex were classified as normal weight.

b Odds ratios adjusted for age and clustering by school.

c Numbers do not add to total because of missing values.

d *P* = .048.

e *P* = .045.

f *P* = .009.

**Table 4:** Adjusted Odds of Being in a Behavioral Category, by Participant Characteristics, Boys (n = 2,083)^a^, School Health Action, Planning and Evaluation System (SHAPES), Ontario, Canada, 2005-2006

Characteristic	High Active-High Sedentary (n = 764) vs High Active-Low Sedentary (n = 213), AOR (95% CI)^b^	Low Active-Low Sedentary (n = 111) vs High Active-Low Sedentary (n = 213), AOR (95% CI)^b^	Low Active-High Sedentary (n = 736) vs High Active-Low Sedentary (n = 213), AOR (95% CI)^b^
**Body mass index^c^**			
Normal weight	1 [Reference]	1 [Reference]	1 [Reference]
Underweight	0.95 (0.59-1.51)	2.63 (1.17-5.91)^d^	2.44 (1.37-4.34)^e^
Overweight	0.91 (0.51-1.63)	1.26 (0.38-4.15)	1.06 (0.50-2.27)
**Perception of body weight**			
Right weight	1 [Reference]	1 [Reference]	1 [Reference]
Overweight	2.17 (1.24-3.79)^e^	3.05 (1.18-7.86)^d^	3.72 (1.90-7.28)^f^
Underweight	1.40 (0.93-2.12)	2.13 (0.94-4.80)	1.48 (0.88-2.50)
**Parents' encouragement of physical activity**			
Do not encourage	1 [Reference]	1 [Reference]	1 [Reference]
Encourage	1.28 (0.80-2.04)	2.52 (1.01-6.27)^d^	0.77 (0.46-1.31)
**Parents' support of physical activity**			
Unsupportive	1 [Reference]	1 [Reference]	1 [Reference]
Supportive	0.56 (0.21-1.54)	0.20 (0.04-0.96)^d^	0.41 (0.15-1.14)
**Participate in intramural teams at school**			
No	1 [Reference]	1 [Reference]	1 [Reference]
Yes	0.99 (0.66-1.47)	0.31 (0.14-0.69)^e^	0.38 (0.23-0.64)^f^
**Participate in varsity teams at school**			
No	1 [Reference]	1 [Reference]	1 [Reference]
Yes	0.82 (0.55-1.22)	0.45 (0.20-0.99)^d^	0.40 (0.25-0.66)^f^
**Participate in team sports outside of school**			
No	1 [Reference]	1 [Reference]	1 [Reference]
Yes	0.76 (0.52-1.11)	0.32 (0.16-0.63)^e^	0.20 (0.13-0.31)^f^

Abbreviations: AOR, adjusted odds ratio; CI, confidence interval.

a Numbers may not add to total because of missing values. All analyses controlled for age, grade level, and school location.

b Numbers may not add to total because of missing values. All analyses controlled for age, grade level, and school location.

c Students who were less than the 5th percentile for BMI by sex were classified as underweight, students who were in the 85th percentile or higher for BMI by sex were classified as at risk of overweight/overweight, and students who were in the 5th to less than the 85th percentile for BMI by sex were classified as normal weight.

d *P* < .05.

e *P* < .01.

f *P* < .001.

**Table 5:** Adjusted Odds of Being in a Behavioral Category, by Participant Characteristics, Girls (n = 1,526)^a^, School Health Action, Planning and Evaluation System (SHAPES), Ontario, Canada, 2005-2006

**Behavioral Characteristic**	**High Active-High Sedentary (n = 344) vs High Active-Low Sedentary (n = 152), AOR (95% CI)^b^**	**Low Active-Low Sedentary (n = 242) vs High Active-Low Sedentary (n = 152), AOR (95% CI)^b^**	**Low Active-High Sedentary (n = 619) vs High Active-Low Sedentary (n = 152), AOR (95% CI)^b^**
**Body mass index^c^**			
Normal weight	1 [Reference]	1 [Reference]	1 [Reference]
Underweight	1.54 (0.81-2.90)	2.64 (1.04-6.75)^d^	1.91 (0.87-4.20)
Overweight	1.58 (0.76-3.29)	1.98 (0.72-5.40)	1.22 (0.49-3.02)
**Perception of body weight**			
Right weight	1 [Reference]	1 [Reference]	1 [Reference]
Overweight	1.82 (1.03-3.18)^d^	1.39 (0.63-3.10)	1.99 (1.01-3.95)^d^
Underweight	1.49 (0.77-2.88)	1.07 (0.40-2.87)	1.12 (0.50-2.51)
**Parents' encouragement of physical activity**			
Do not encourage	1 [Reference]	1 [Reference]	1 [Reference]
Encourage	1.03 (0.53-2.02)	1.20 (0.55-2.61)	0.69 (0.37-1.30)
**Parent support of physical activity**			
Unsupportive	1 [Reference]	1 [Reference]	1 [Reference]
Supportive	0.81 (0.28-2.36)	1.09 (0.34-3.50)	0.92 (0.33-2.59)
**Participate in intramural teams at school**			
No	1 [Reference]	1 [Reference]	1 [Reference]
Yes	1.14 (0.67-1.95)	0.38 (0.18-0.83)^d^	0.48 (0.25-0.92)^d^
**Participate in varsity teams at school**			
No	1 [Reference]	1 [Reference]	1 [Reference]
Yes	0.90 (0.52-1.55)	0.50 (0.24-1.05)	0.56 (0.30-1.04)
**Participate in team sports outside of school**			
No	1 [Reference]	1 [Reference]	1 [Reference]
Yes	0.34 (0.21-0.57)^f^	0.10 (0.05-0.18)^e^	0.09 (0.05-0.15)^f^

Abbreviations: AOR, adjusted odds ratio; CI, confidence interval.

a Numbers may not add to total because of missing values. All analyses controlled for age, grade level, and school location.

b Odds ratios adjusted for age, grade level, father's physical activity level, mother's physical activity level, close friends' physical activity level, smoking status, school location, clustering by school, and all other variables in the table.

c Students who were less than the 5th percentile for BMI by sex were classified as underweight, students who were in the 85th percentile or higher for BMI by sex were classified as at risk of overweight/overweight, and students who were in the 5th to less than the 85th percentile for BMI by sex were classified as normal weight.

d *P* < .05.

e *P* < .01.

f *P* < .001.
